# Circulating tumour markers can define patients with normal colons, benign polyps, and cancers

**DOI:** 10.1038/bjc.2011.230

**Published:** 2011-06-28

**Authors:** R Mead, M Duku, P Bhandari, I A Cree

**Affiliations:** 1 Department of Gastroenterology and Translational Oncology Research Centre, Queen Alexandra Hospital, Portsmouth PO6 3LY, UK

**Keywords:** colorectal cancer, circulating, DNA, plasma, CEA, tumour marker

## Abstract

**Background:**

Early diagnosis represents the best opportunity for cure of colorectal cancer. Current screening programmes use faecal occult blood testing for screening, which has limited sensitivity and poor specificity.

**Methods:**

In this study we looked at a series of previously described diagnostic markers utilising circulating free DNA (cfDNA), with a preparation method allowing small DNA fragments to be isolated. The Circulating free DNA was isolated from samples obtained from 85 patients, including 35 patients without endoscopic abnormality, a group of 26 patients with benign colorectal adenomas, and 24 patients with colorectal carcinomas. In each case, polymerase chain reaction (PCR) was performed for Line1 79 bp, Line1 300 bp, Alu 115 bp, Alu 247 bp, and mitochondrial primers. In addition, carcinoembryonic antigen (CEA) was measured by ELISA. Each marker was analysed between normal, polyp, and cancer populations, and the best performing analysed in combination by logistic regression.

**Results:**

The best model was able to discriminate normal from populations with adenoma or carcinoma using three DNA markers and CEA, showing an area under the receiver operator characteristic (ROC) curve of 0.855 with a positive predictive value of 81.1% for polyps and cancer diagnosis.

**Conclusion:**

These circulating markers in combination with other markers offer the prospect of a simple blood test as a possible secondary screen for colorectal cancers and polyps in patients with positive faecal occult blood tests.

Colorectal cancer is the second most common cause of death from cancer in the United Kingdom, and the third most common cancer overall. Approximately 100 new cases of colorectal cancer are diagnosed each day, and 16 000 people die from this disease each year in the United Kingdom (Cancer Research UK, 2011). The disease biology is well understood with a sequence of adenoma, increasing dysplasia, and carcinoma commonly demonstrated (Allen, 1995; Vogelstein and Kinzler, 2004). Death is secondary to widespread metastasis, particularly involving the liver, which does not occur in dysplasia and early cancer restricted to the bowel wall. Therefore, early diagnosis represents the best opportunity for cure.

The United Kingdom is fortunate in having an established bowel cancer screening programme. However, this relies upon patients sampling their own faeces up to nine times with a guaiac-based occult blood test (fOBT). Although practical and affordable, this system leaves room for improvement (Vernon, 1997; van Dam *et al*, 2010), with initial UK compliance rates at 56.8% (http://www.cancerscreening.nhs.uk/bowel/), and considerable variation worldwide (18–90%) (Ore *et al*, 2001; Federici *et al*, 2005; Fenocchi *et al*, 2006; Parente *et al*, 2009). The fOBT also has a low estimated sensitivity for cancer (40.58%), and even lower sensitivity for significant dysplastic polyps (5.00%) (http://www.cancerscreening.nhs.uk/bowel/). Current treatment options offer cure in 90–100% of patients with dysplasia and early cancers (Cancer Research UK, 2011); however, identification of these often asymptomatic patients remains challenging.

In cancer patients, the presence of circulating free DNA (cfDNA) in the blood has been well described (Boni *et al*, 2007; Tomita *et al*, 2007; Catarino *et al*, 2008; Kolesnikova *et al*, 2008; Sunami *et al*, 2008; Toth *et al*, 2009; Yoon *et al*, 2009; Ellinger *et al*, 2009a, 2009b; Dobrzycka *et al*, 2010; Liggett *et al*, 2010; van der Drift *et al*, 2010), and is considered to be a derivative of increased and abnormal apoptotic pathways in the cancerous lesions (Stroun *et al*, 2001; Tuaeva *et al*, 2008). The abnormal DNA degradation or secretion (Stroun *et al*, 2001) leads to increased DNA levels and differing DNA fragmentation, readily identifiable with standardised and affordable molecular biology techniques (Drew, 2007).

Studies of cfDNA in both plasma and serum have been reported with promising markers identified (Boni *et al*, 2007; Tomita *et al*, 2007; Catarino *et al*, 2008; Kolesnikova *et al*, 2008; Sunami *et al*, 2008; Toth *et al*, 2009; Yoon *et al*, 2009; Ellinger *et al*, 2009a, 2009b; Dobrzycka *et al*, 2010; Liggett *et al*, 2010; van der Drift *et al*, 2010). However, differing levels of cfDNA are found between and within experiments using serum and plasma, and the optimal method for processing blood is still debated (Kopreski *et al*, 1997; Chiu *et al*, 2001; Taback *et al*, 2004; Umetani *et al*, 2006b; Board *et al*, 2008; Xue *et al*, 2009; Yoon *et al*, 2009; Thierry *et al*, 2010; van der Vaart and Pretorius, 2010). DNA purification techniques have recently improved, allowing purification of small (20+ bp) cfDNA fragments. Small fragment DNA has been suggested as an important component of circulating DNA in cancer patients (Rago *et al*, 2007; Muller *et al*, 2008; Thierry *et al*, 2010), and has not previously been included in many studies isolating >100 bp fragments (Wang *et al*, 2004; van der Vaart and Pretorius, 2010).

Raised levels of cfDNA have been identified in patients with inflammatory conditions and in patients admitted to hospital for medical treatment suggesting increased apoptosis in these non-neoplastic conditions (Harbeck *et al*, 1975; Holdenrieder *et al*, 2001; Chang *et al*, 2002; Lam *et al*, 2004; Margraf *et al*, 2008; Perego *et al*, 2008). Raised cfDNA levels cannot therefore be regarded as specific to cancer, although different size distributions have been noted in cancer patients (Wang *et al*, 2003; Ellinger *et al*, 2008). As cfDNA lacks specificity, it is potentially useful to combine its assessment with other markers. Carcinoembryonic antigen (CEA) is used clinically as a marker of colorectal cancer progression, but alone is not useful as a diagnostic marker (Locker *et al*, 2006). Nevertheless, we felt it useful to include CEA in our analysis as it is readily available and could be assessed for its contribution to the multimarker model.

The aim of this study was to confirm the presence of previously described diagnostic markers in sera (Umetani *et al*, 2006a, 2006c; Diehl *et al*, 2008; Sunami *et al*, 2008), test the addition of mitochondrial and small fragment DNA markers (Diehl *et al*, 2005; Lin *et al*, 2008), and optimise them for analysis in plasma (Chiu *et al*, 2001; Kirsch *et al*, 2008), a source less likely to be contaminated from leukocytes (Lee *et al*, 2001; Jung *et al*, 2003; Taback *et al*, 2004; Yoon *et al*, 2009; Thierry *et al*, 2010; van der Vaart and Pretorius, 2010). With a cleaner source, and purification including small fragment DNA, we aimed to identify changes in plasma from patients without known inflammatory conditions undergoing either colonoscopy to remove large precancerous lesions or curative surgery for colon cancer.

## Patients and Methods

### Patient selection

Participants were screened for inflammatory conditions and previous cancer by history, patient letters, and recent blood tests before consent. Patients undergoing endoscopic investigation without significant clinical findings were included as a ‘normal’ control group. Blood samples were taken after the normal investigation. Patients undergoing therapeutic endoscopy for large benign polyps had samples taken before endoscopic intervention commenced. This group included patients with low-grade dysplasia, high-grade dysplasia, and polyp cancers, and grouped according to the highest-grade polyp. Patients with established colorectal cancer were admitted to surgical wards before attempted surgical cure, and samples were taken as part of the preoperative work-up.

### Sample processing

Venous blood was transferred in BD Vacutainer EDTA tubes (Becton Dickinson, Oxford, UK) and processed within 1–4 h (Lee *et al*, 2001; Xue *et al*, 2009). Samples were mixed with same volume of phosphate-buffered solution before layering over Histopaque-1077 (Sigma-Aldrich Ltd, Poole, UK) and centrifuging for 30 min at 600 **g**. The clear plasma above the separated cellular layer was carefully removed without disturbance to the cellular layer. Further centrifugation at 400 **g** for 7 min was carried out before removing the upper layer of plasma with a sterile pastette (without disturbing the lower levels). Samples were divided into 2 ml aliquots and stored at −80 °C.

Before DNA purification, plasma samples were thawed and spun at 11 000 **g** for 3 min. DNA was purified with a modified high-yield Nucleospin Plasma XS protocol, using 960 *μ*l of plasma according to the manufacturer's instructions (Macherey-Nagel, DÜren, Germany). DNA was stored at −20 °C in 30 *μ*l of elution buffer, and thawed only once.

### Polymerase chain reaction

Polymerase chain reaction (PCR) was performed on an AB7500 PCR machine (Applied Biosystems Inc., Foster City, CA, USA). SYBR green master-mix and primers (Applied Biosystems) were optimised for Line1 79 bp (Diehl *et al*, 2008), Line1 300 bp (Sunami *et al*, 2008), Alu 115 (Umetani *et al*, 2006c), Alu 247 (Umetani *et al*, 2006c), and mitochondrial primers (Lin *et al*, 2008). DNA was diluted 1 : 40 before triplicate evaluation across five separate primer plates. A single standardised solution of lymphocyte DNA was used as a standard curve reference (identical across all five plates) (Diehl *et al*, 2008; Sunami *et al*, 2009). Mean values across triplicates were used for further analysis.

### Total DNA analysis

DNA quantification utilised PCR of the repetitive Line1 79 bp fragment, as described by Diehl *et al* (2008). Essentially, the PCR reaction was performed in triplicate against a common genomic DNA standard curve, and 5 *μ*l of 1 : 40 DNA suspension was added to 20 *μ*l of SYBR green with 400 *μ*mol Line1 79 bp primers. Total DNA in ng ml^–1^ was calculated using the internal AB7500 standard software algorithm. Cycling conditions were 2 min at 50 °C, 10 min at 95 °C, and 30 cycles of 95 °C for 15 s, and 60 °C for 1 min. The assay has an intraassay coefficient of variation (CoV) of 8.6% and the interassay CoV was 5.6%.

### CEA analysis

The CEA analysis was performed using a Beckman Coulter Unicel DXL 800 machine (CEA2 assay) with a CEA2 kit (CEA Access, Beckman Coulter ref 33200, High Wycombe, UK). This is an immunoassay with Lumi-Phos 530 (Lumigen PPD (4-methoxy-4-(3-phosphatephenyl)spiro[1,2-dioxetane-3,2′-adamantane], disodium salt) as the fluorescent marker, and alkaline phosphatase as the linked enzyme. The reaction produces fluorescence at 530 nm, which is quantitatively assayed.

Serum is the usual source of CEA, and with half-strength plasma there is no recognised clinical cutoff. The results have therefore been used quantitatively as part of a regression model, recognising this limitation.

### Ethical approval and data analysis

Local ethical approval was undertaken, and samples stored according to a local tissue bank protocol. Paired data were analysed using Mann–Whitney *U*-test, and multiple group analysis by Kruskal–Wallis test. Receiver operator characteristic (ROC) analysis was used to assess for diagnostic suitability, and logistic regression analysis to analyse marker combination. Both were undertaken using SPSS ver. 16.0 (IBM United Kingdom Limited, Portsmouth, UK) with statistical advice. As an increased type I error is associated with multiple marker analysis, a 10-fold Bonferroni correction has been applied, reducing the significant *P*-value to 0.005.

## Results

### Study population

The study population comprised 85 patients, including 35 patients without endoscopic abnormality, a group of 26 patients with benign colorectal adenomas, and 24 patients with colorectal carcinomas. The reference ‘normal’ group of 35 patients, including 15 men, was chosen as representing a typical population attending for endoscopic investigation but with no detectable abnormality (Table 1). The mean age of this group was 54.1 years (range 24–80 years). Significant intercurrent diagnoses in this group included: osteoarthritis, ischaemic heart disease, pernicious anaemia, and peptic ulcer.

The benign colorectal polyp group of 26 patients, including 14 men, had a mean age of 70.2 years (range 56–85 years). From these patients, 29 polyps were removed, with mean size 54.3 mm (range 5–200 mm). Of these polyps, 18 showed low-grade dysplasia (LGD), and had a mean size of 56.1 mm (range 5–200 mm), whereas 11 polyps showed high-grade dysplasia (HGD), with mean size 52.5 mm (range 28–100 mm). Significant residual polyp remained in three patients, and a further blood sample taken from these patients is included in the analysis.

The group of cancer patients included 19 men and had a mean age of 71.5 years (range 49–87 years). There were four patients with polyp cancers, with mean size 41.7 mm (range 25–40 mm), but the majority had biopsy-proven colorectal carcinomas, with pathological staging as: pT0 × 5 (4 polyps, 1 post radiotherapy); pT2 × 4; pT3 × 14; pT4 × 1.

In total, both polyp and cancer populations included 50 patients, and 53 plasma samples, with a mean age of 71.1 years (49–87 years).

### Individual marker utility

Each marker was analysed between normal, polyp, and cancer populations (Table 1). There was an increase in the mean values of all markers with increasing pathological grade, although not all these differences were statistically significant (Table 2). There was no significant correlation with age in the normal population.

**Figure 1:**
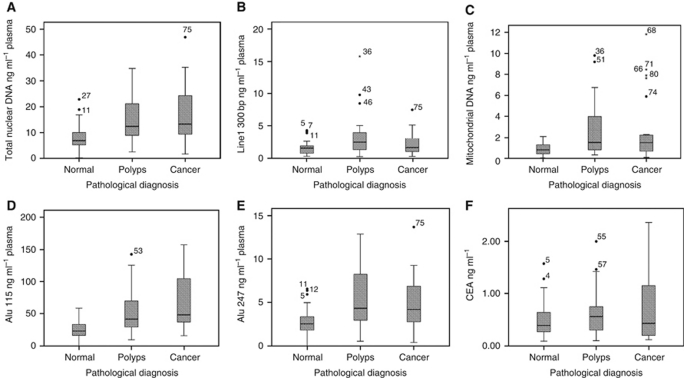
Box plots for each of the DNA markers measured showing medians as bars and interquartile range as box. (**A**) Total DNA expressed as the concentration of the repetitive Line1 79 bp DNA fragment (*P*<), (**B**) Line1 300 bp fragment, (**C**) mitochondrial DNA (*P*<), (**D**) Alu 115 fragment, (**E**) Alu 247 bp fragment, and (**F**) CEA.

When compared with normal controls, patients with benign polyps had significantly raised levels of total DNA (*P*<0.001), mitochondrial DNA (*P*=0.001), and Alu 247 bp fragment (*P*=0.001, Table 2 and Figure 1). These markers also show the best ROC curves for diagnostic testing. The total DNA (Line1 79 bp) was the best single marker (ROC=0.756, Table 3).

**Figure 2:**
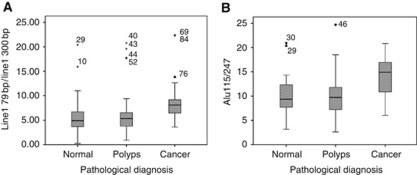
Box plots for ratios of the DNA markers measured showing medians as bars and interquartile range as box. (**A**) Line1 79/300 ratio and (**B**) Alu 115/247 bp ratio.

When compared with normal controls, patients with colon cancer had significantly raised levels of the fragmentation ratios Line1 79/300 (*P*=0.001) and Alu 115/247 (*P*<0.001) (Table 2 and Figure 2). The total DNA and the Alu 247 bp markers also remained significantly different, with all four markers showing high ROC values. The Alu 115/247 ratio was found to be the best single ratio (ROC=0.772, Table 3) for distinguishing cancer from normal in this series.

Comparison of normal with all neoplasia patients showed total DNA (*P*<0.001), mitochondrial DNA per ml (*P*<0.001), and Alu 247 bp (*P*<0.001) markers to be highly statistically significant. The best single marker for diagnosis of neoplasia in this population was total DNA (ROC=0.756, Table 3). There were occasional outlier results: a single patient with a LGD polyp represented an extreme outlier, with a total DNA level of 206 ng ml^–1^. This patient was removed from the mean data, but is included in all other analyses.

Carcinoembryonic antigen is currently the only circulating marker in use for colon cancer follow-up, although it has again been shown in this series that it is a poor diagnostic marker for colonic neoplasia, with no significant difference between the populations, and low ROC values (best ROC=0.596). However, its clinical role is in those with high levels already diagnosed with colonic cancer and therefore it was included as part of a panel of diagnostic markers as described below.

### Logistic regression analysis

The best performing markers were combined and analysed in combination by logistic regression. The predicted probabilities of diagnosis generated a ‘combination marker’ ROC curve for all three categories (Table 4).

**Figure 3:**
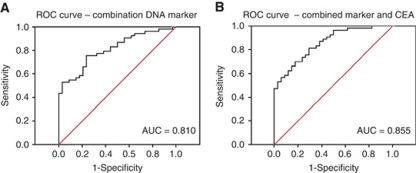
(**A**) ROC curve for the optimal combination marker-derived logistic regression from three markers (total nuclear DNA, Alu 247, and mitochondrial DNA), and (**B**) with CEA forced into the model.

The best ROC curve to discriminate normal from neoplasia populations, with four DNA markers (Line1 79 bp, Alu 247 bp, mitochondrial DNA, and Alu 115 bp), showed ROC curve of 0.810 (Figure 3A). Combining CEA levels and the combination DNA marker demonstrated the possibilities of a multiple-target blood test and showed ROC curve of 0.855 (Table 4 and Figure 3B). The final test had a positive predictive value (PPV) of 81.1% for polyps and a negative predictive value (NPV) of 73.5% (sensitivity 83% and specificity 72%) for early cancer diagnosis. If the threshold was altered to achieve the best NPV (97.1%), the PPV was 49.1%.

## Discussion

Colorectal cancer carries a high mortality in the United Kingdom, and efforts to diagnose the disease at an earlier stage are regarded as key to reduce associated deaths (Richards, 2009). Screening in colorectal cancer is especially challenging with the diagnosis of precancerous adenomatous polyps regarded as essential for prevention, and representing a more difficult diagnostic target.

This study demonstrates highly significant differences between a ‘normal’ population, a population with adenomatous polyps, and a population with colonic cancer. Quantities and patterns of circulating DNA differ significantly between the groups, with total quantities of nuclear and mitochondrial DNA increasing with pathological grade, and fragmentation patterns changing between normal, adenomatous, and cancer populations. Individual markers demonstrate good diagnostic tests, and in combination provide an improved test for both polyps and cancer (ROC=0.810). To our knowledge, this is the first study to demonstrate these circulating DNA changes in colonic polyps and cancers, offering the prospect of improved diagnosis of neoplasia in colorectal cancer screening with a simple blood test. Although the test lacks specificity for colorectal cancer, it could be particularly useful as a triage test for patients with a positive faecal occult blood test. Those with cfDNA below a threshold defined by the ROC curve could be spared the risk and cost of colonoscopy.

Although a larger population would be desirable, these results arise in a clinically very relevant population with other results providing good support for our findings. Strikingly similar DNA levels in serum, from a study of more advanced colon cancers, showed a healthy control group with an almost identical median total DNA level of 7.7 ng ml^–1^ (in current study, 6.86 ng ml^–1^), and cancer patients with 35.8 ng ml^–1^ (in current study, 14.58 ng ml^–1^). A combined ROC score in cancer cases was 0.92 (Flamini *et al*, 2006).

Although total DNA levels are significantly different across pathological grade, neither polyp size nor estimated surface area showed significant correlation in both the total polyp population (*P*=0.28) and the larger category of LGD polyps (*P*=0.24). Accurate sizing of polyps is however difficult, with lateral and outward growth contributing to an overall volume; therefore, although we have found no correlation, it is difficult to be entirely confident that size of polyp is not relevant to cfDNA levels.

Mitochondrial DNA, with multiple copies in each cell, has been proposed to provide a more accurate marker in the analysis of low quantities of circulating DNA. In agreement with this, our study shows highly significant differences between the groups, with a rise in benign and cancer groups. However, in logistic regression analysis it does not add to the accuracy obtained with standard markers, and other studies have also reported both high and low levels in cancer (Huang *et al*, 2009; Xia *et al*, 2009; Hosgood *et al*, 2010).

Total DNA and Alu 247 fragment results have now been demonstrated to be consistent across studies in both noninflammatory normal patients and three types of cancer. We believe that they offer the prospect of a broad neoplasia screening test in noninflammatory cancers. This approach would be highly attractive to patients, although taxing for physicians in an asymptomatic population, and will require specificity for cancer type. This may be available from other tests, possibly on the same blood sample. For example, in our study the 300 bp marker does not perform well, but has shown promise in breast cancer (Sunami *et al*, 2008). This difference may help localise gastrointestinal cancers from other cancer types when Alu 247 is raised. This interesting finding may relate to the biological handling of cfDNA from the digestive system before it enters the normal circulation (Gauthier *et al*, 1996; Taback *et al*, 2006), and may give the Alu 247 bp fragment a degree of specificity for colorectal neoplasia. Alternatively, specificity may be achieved by using additional PCR markers such as methylated septin 9 or ELISA-based assays with colon-cancer-specific-2 or TIMP 1 (Holten-Andersen *et al*, 2002; Leman *et al*, 2008; deVos *et al*, 2009; Church *et al*, 2010) proteins, allowing combinations similar to the CEA results in our study, and offering the prospect of a more specific blood test screen for colorectal cancer. In our study, CEA did not add greatly to the AUROC, and the results are included largely to show that this marker, even in combination with others, has little diagnostic value. Nevertheless, the addition of protein and DNA markers may give greater accuracy of such tests and should be considered further.

It may well be that identification of normal patients not requiring other screening interventions will be the ultimate role of cfDNA markers. The Alu 247 fragment as the best single performer in our study, previously showed significant findings discriminating colon cancer, ampullary cancer (Umetani *et al*, 2006c), and breast cancer patients (Umetani *et al*, 2006a), and has shown early promise as a universal marker in this role.

Reservations regarding the utility of DNA markers in the normal population have been expressed, with an ovarian study including a population of patients admitted to hospital for treatment with associated benign diseases. Results from this group show raised cfDNA levels at 16 ng ml^–1^ (Chang *et al*, 2002), and a reduced diagnostic accuracy. However, these ‘benign’ conditions included patients admitted to hospital with infections, autoimmune disease, postorgan transplant, post-trauma, AIDS, and asthma. All of these conditions are likely to be associated with active inflammation and increased apoptosis. Furthermore, these conditions cause proven increases in circulating DNA (Lui and Dennis, 2002; Lam *et al*, 2003, 2004; Moreira *et al*, 2010), and inflammation in a study of circulating DNA histones showed a clear correlation with DNA levels (Holdenrieder *et al*, 2001). Raised cfDNA has been noted in rheumatoid disease, blamed on antibody-bound cfDNA (Zhong *et al*, 2007). Such severely ill patients are unlikely to be represented in large numbers within the population attending screening visits and would be rapidly removed from the test pool. We therefore believe that confounding factors can be assessed by clinical history, allowing study of clinically relevant populations that would show clear differences in DNA levels even in precancerous conditions. Other studies suggest that physiological changes with age and even menstruation, driven by apoptosis within the endometrium, are not associated with changes in cfDNA (Polcher *et al*, 2010).

In conclusion, cfDNA markers offer an interesting prospect for a blood test screen in cancer, with our results showing the possibilities in early colon cancer and precancerous polyps. We have identified a combination marker in our population able to discriminate ‘normal’ patients with common medical problems from patients with benign polyps and operable cancers, although this requires validation in a larger independent series.

## Figures and Tables

**Table 1:** Normal population (*n*=35) indications for endoscopy

**Endoscopy indication**	**Number**
Dysphagia	5
Dyspepsia	10
Ulcer follow-up	1
Haematemesis	2
Abdominal pain	8
Change in bowel habit	7
Family history of cancer	1
Anaemia	5
Polyp follow-up	1
Melaena	1

Some patients had multiple indications.

**Table 2:** Mean values for circulating markers by pathology

	**Age, years**	**CEA ng ml^–1^**	**Total nuclear DNA ng ml^–1^ plasma**	**Mitochondrial DNA ng ml^–1^ plasma**	**Line1 79/ Line1 300**	**Alu 115/247**	**Line1 300 ng ml^–1^ plasma**	**Alu 247 ng ml^–1^ plasma**
Normal	54.1	0.49	7.96	0.90	6.67	10.01	1.54	2.86
Polyps	70.2	0.65	15.04	2.68	7.76	11.89	3.25	5.56
Cancer	71.5	1.06	30.09	4.05	9.11	14.02	3.56	7.51

Abbreviation: CEA=carcinoembryonic antigen.

**Table 3:** Significance testing of markers in populations with normal endoscopy, polyps, or cancer

	**CEA ng ml^–1^**	**Total nuclear DNA ng ml^–1^ plasma**	**Mitochondrial DNA ng ml ^–1^plasma**	**Line1 79/ Line1 300**	**Alu 115/247**	**Line1 300 ng ml^–1^ plasma**	**Alu 247 ng ml^–1^ plasma**
Polyps *vs* normal	0.254	*P*<0.001	0.001	0.721	0.879	0.007	0.001
Cancer *vs* normal	0.396	0.001	0.017	0.001	*P*<0.001	0.174	0.004
Polyps and cancer *vs* normal	0.234	*P*<0.001	*P*<0.001	0.033	0.043	0.014	*P*<0.001

Abbreviation: CEA=carcinoembryonic antigen.

Mann–Whitney *U-*test, Bonferroni correction applied, significance level *P*=0.005.

**Table 4:** ROC curve values for each marker and combination marker by pathology

	**CEA ng ml^–1^**	**Total nuclear DNA ng ml^–1^ plasma**	**Mitochondrial DNA ng ml^–1^ plasma**	**Line1 79/ Line1 300**	**Alu 115/247**	**Line1 300 ng ml^–1^ plasma**	**Alu 247 ng ml^–1^ plasma**	**Combined marker**
Polyps *vs* normal	0.596	0.756	0.743	0.512	0.511	0.691	0.731	0.797
Cancer *vs* normal	0.574	0.757	0.675	0.759	0.772	0.594	0.716	0.863
Polyps and cancer *vs* normal	0.586	0.756	0.713	0.624	0.629	0.647	0.724	0.810

Abbreviations: CEA=carcinoembryonic antigen; ROC=receiver operator characteristic.
